# A review of recent advances in the decontamination of mycotoxin and inactivation of fungi by ultrasound

**DOI:** 10.1016/j.ultsonch.2021.105755

**Published:** 2021-09-17

**Authors:** Motahareh Hashemi Moosavi, Amin Mousavi Khaneghah, Fardin Javanmardi, Milad Hadidi, Zahra Hadian, Shima Jafarzadeh, Elcin Huseyn, Anderson S. Sant'Ana

**Affiliations:** a Department of Food Science and Technology, National Nutrition and Food Technology Research Institute, Faculty of Nutrition Sciences and Food Technology, Shahid Beheshti University of Medical Sciences, Tehran, Iran; b Department of Food Science and Nutrition, Faculty of Food Engineering, University of Campinas, Campinas, SP, Brazil; c Department of Organic Chemistry, Faculty of Chemical Sciences and Technologies, University of Castilla-La Mancha, 13071 Ciudad Real, Spain; d Food Technology Division, School of Industrial Technology, Universiti Sains Malaysia, Penang, Malaysia; e Research Laboratory of Intelligent Control and Decision Making Systems in, Industry and Economics, Azerbaijan State Oil and Industry University, Azerbaijan

**Keywords:** Microorganisms, Decontamination, Ultrasound, Mycotoxin, Food contaminant

## Abstract

Innovative technologies for the pasteurization of food products have increased due to the global demand for higher-quality food products. In this regard, the current article aimed to provide an overview regarding the latest research on US application in the decontamination of fungi in food products and highlight the parameters influencing the effectiveness of this method. Therefore, the related article with inactivation of fungi and mycotoxins by ultrasound among last four years (2018–2021) by using terms such as 'mycotoxin,' 'inactivation,' 'ultrasound,' 'decontamination' among some international databases such as PubMed, Web of Science, Embase and Google Scholar“ was retrieved. Ultrasound (US) is considered a non-thermal decontamination method for food products. In US, the release of energy due to the acoustic phenomenon destroys microorganisms. This technology is advantageous as it is inexpensive, eco-friendly, and does not negatively affect food products' food structure and organoleptic properties. The influence of the US on food structure and organoleptic properties dramatically depends on the intensity and energy density applied In addition, it can preserve higher levels of ascorbic acid, lycopene, and chlorophyll in sonicated food products. The treatment conditions, including frequency, intensity, duration, temperature, and processing pressure, influence the effectiveness of decontamination. However, US displays synergistic or antagonistic effects on bacteria, yeasts, molds, and mycotoxins when combined with other types of decontamination methods such as chemical and thermal approaches. Thus, further research is needed to clarify these effects. Overall, the application of US methods in the food industry for decreasing the microbial content of food products during processing has been applied. However, the use of US with other techniques needs to be studied further.

## Highlights

• Ultrasound (US) is an emerging technology for fungal decontamination in foods.
• Frequency, intensity, duration, temperature, and pressure impact US effectiveness.
• Combination of ultrasound with other techniques improves decontamination.

## 1 Introduction

The demand for healthier food products has increased global concerns regarding food spoilage. Fungi are among the most predominant food pathogens in various food products, including milk, cereals, meat, vegetables, edible oils and fruits [1], [4], [18]. Fungal growth in food products can lead to off-flavors, acidification, disintegration, and discoloration; moreover, it can lead to the formation of toxic metabolites called mycotoxins, which exhibit immunosuppressive, carcinogenic, mutagenic, and teratogenic effects [4], [25]. The primary source of food contamination is the raw materials that become polluted during harvest, transportation, and storage [20]. Despite effective measures for monitoring fungal contamination at first, food safety control can be achieved by decreasing the number of pathogenic microorganisms through proper food processing conditions [17].

Traditional food preservation methods can reduce the number of microorganisms to safe levels. However, using these methods leads to the loss of beneficial food ingredients such as heat-sensitive vitamins, aromatic compounds, and color pigments [50]. Recently, non-thermal decontamination technologies have attracted increased attention. Non-thermal decontamination technologies, including high hydrostatic pressure, cold plasma, UV light, pulsed electric field, and ultrasound (US), can effectively destroy microorganisms with negligible adverse effects on the nutritional value and sensory properties of food materials [40], [12]. These methods apply mild temperature conditions and shorter processing time, which retain the flavor, enhance the shelf life and inactivate enzymes [7], [8], [11]. Thus, these approaches are attractive for producing high-quality and fresh products [50].

Ultrasound has been the research subject as a non-thermal and ecofriendly technique [40]. Ultrasound has several advantageous properties. For example, it decreases the use of fossil fuels to provide energy during food processing, including drying and heating; it decreases the amount of water consumed; it enhances the productivity; and it retains the nutrients of the product [7], [44]. Ultrasound waves (frequency >20 kHz) with specific intensity and amplitude are used for inactivating microorganisms in food products. The cavitation phenomenon is the main factor behind microbial destruction [33]. In this process, the cavitation bubbles are formed through cycles of pressure created by high-intensity ultrasound. The bubbles grow over several compression/rarefaction cycles until they reach an unstable size, and the cavitation bubble undergoes an implosive collapse, leading to the release of energy. The released energy induces very high shear forces by creating high-temperature and pressure conditions (5000 K and 5000 atm), leading to the structural destruction of many microorganisms [22], [35].

The related article with inactivation of fungi and mycotoxins by ultrasound among last four years (2018–2021) by using terms such as 'mycotoxin,' 'inactivation,' 'ultrasound,' 'decontamination' among some international databases such as PubMed, Web of Science, Embase and Google Scholar“ was retrieved and aimed to evaluate the latest research on US application in the decontamination of fungi in food products and highlight the parameters influencing the effectiveness of this method.

## 2 Overview of US processing

**Fig. 1:**
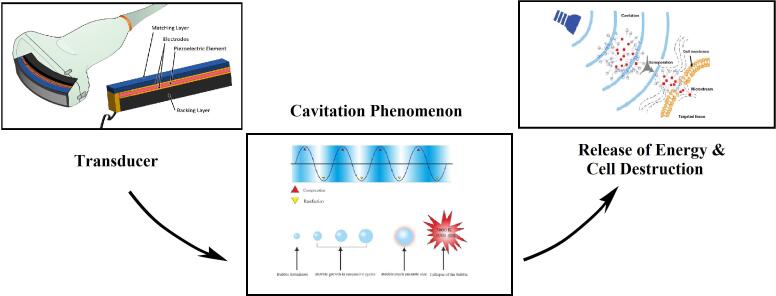
Mechanism of action for Ultrasound [51].

Due to the many applications of US in the food industry, the use of this technology is growing. The use and effectiveness of US in the food industry depend on the frequency (20 kHz to 3 MHz), acoustic intensity (1–10,000 W/cm^2^), and physical state of food (liquid, solid, and gas) [22]. Low-frequency high-intensity US waves (intensities in the range of 10–1000 W/cm at frequencies between 18 and 100 kHz) indicate the low energy that can be used to investigate products, including their structure, composition, and physical status because they can retain the physical and chemical structures of products [36]. High-frequency low-intensity US covers the frequencies higher than 100 kHz at intensities below 1 W/cm, which is known as power US. It is used in cleaning, extraction, chemical reactions, emulsification, microbial and enzyme inactivation, and food processing (e.g., defoaming, degassing, and drying) [43]. The inactivation of microorganisms by cavitation depends on the medium in which ultrasound is applied. During rarefaction, the negative pressure results in a pulling effect, which leads to bubble nucleation. To create bubbles/cavities, the cohesive force between solvent molecules needs to be overcome. The formed bubbles grow by absorbing energy through these cycles. After energy can no longer be absorbed, the microbubbles collapse, and a considerable amount of energy is released to the medium (Fig. 1). This phenomenon leads to the chemical and physical transformation of the structure of pathogenic fungi and bacteria, which leads to their death [36].

## 3 Ultrasound for fungal inactivation

Ultrasound application in the food industry is highlighted as a substitute for pasteurization or other decontamination methods due to its low temperature and fewer effects on the organoleptic properties of food ingredients [42]. Several theories regarding the mechanism of US in various biological systems have been proposed; three procedures are considered for this phenomenon, including thinning of cell membranes, localized temperature, and generation of free radicals [43]. Cavitation is categorized into two types: stable cavitation and transient cavitation. In stable cavitation, bubbles do not collapse. Instead, bubble vibration exerts a twisting and rotational motion, creating a stream of microcurrents on the sonic field. This microstreaming phenomenon creates forces and strains that lead to cell membrane disruption and cell wall breakdown [9]. Li et al. [23] reported that the combined use of a bath US (300, 400, and 500 W) and hot water at 55 °C for 10 min reduced the colony diameter of *Rhizopus stolonifer*. The results of scanning electron microscopy showed that the leakage of nucleic acid and proteins increased and caused changes in the cell membrane of *R. stolonifer* [23]*.* By contrast, transient cavitation gives rise to the collapse of bubbles, which generates harsh temperature and pressure conditions, resulting in the formation of an active site for chemical reactions and, more importantly, physical damage to the microorganism’s structure [36]. The imploded cavity causes an increase in temperature and pressure, which leads to the thermal decomposition of water molecules and the production of hydroxyl free radicals and hydrogen atoms [38]. Cavitation creates three active sites for chemical interactions. The first site is a hot interior of bubbles with an estimated temperature and pressure of 5200 ± 650 K and 500 atm, respectively, the primary origin of highly reactive radicals. The second site is the shell of microbubbles with a temperature of around 2000 K during bubble collapse [49]. The third site is a bulk solution around the bubbles [36]. Hydrogen and hydroxide are the primary radicals from water molecules in the food structure that break the fungal and bacterial DNA by bonding with the sugar-phosphate site of DNA and destroy them. In addition, the antibacterial effect of hydrogen peroxide eases the disinfection procedure [36], [43]. However, according to Dolas et al., depolymerization of enzymes is the primary mechanism in enzyme inactivation [10]. Enzyme depolymerization may occur due to the cavitation effect or the binding of free radicals with enzymes, which leads to instability of enzymes and loss of their structures. These mechanical, thermal, and chemical effects induce stress to microbes, cause destruction of microbial cell structure, and inactivate key enzymes [32]. Liu et al. [26] reported that A 550 W ultrasonic processor (using a 13 mm probe) with a power intensity of 2.2–11 W/cm^2^ and sonication time range of 10–50 min affected mycotoxin degradation. The results showed that deoxynivalenol (DON) was more stable in US compared with aflatoxin B_1_ (AFB_1_), zearalenone (ZEN), and ochratoxin A (OTA). At a duty cycle of 25%, the degradation and metamorphism rates were higher than 95% for all types of mycotoxins(AFB_1_, DON, ZEN, and OTA) [26]. In another study, Liu et al. [26] used a 550 W power ultrasonic with a 13-mm probe. The frequency used for this study was 20 kHz. They found that 85.1% of AFB_1_ was degraded after 80 min of US exposure with a power intensity of 6.6 W/cm^2^ [27].

## 4 Factors affecting fungal inactivation

The efficacy of US treatment differs based on various parameters. The amplitude of ultrasonic waves, frequency, treatment temperature, and exposure/contact time are influential factors [47]. A previous study demonstrated that specific temperature, exposure time, and higher amplitude of US waves could increase the inactivation level [15]. Generally, a temperature above 50 °C is better for microorganism destruction [15]. In fruits and vegetables, a frequency range between 20 kHz and 40 kHz, treatment duration of 1–60 min, and temperature of 20 °C to 40 °C are much more effective [31]. Agnieszka Starek et al. used a 750 W power sonication (20 kHz, 46- and 58 μm amplitude, with a 19 mm diameter probe) to induce microorganisms (mesophilic aerobic) in tomato juice. They found that a treatment intensity of 40 W/cm and a duration of 10 min significantly decreased the number of microorganisms in tomato juice by 2.8–5.4 CFU/g compared to untreated tomato juice [45]. In another study, the reduction of microorganisms in a strawberry was achieved with sonication for 3 min at 40 kHz by 17-fold compared to the control group that strawberries were dipped in distilled water [28]. Also, a combination of US and ozone was most effective in reducing bacterial survival (98%).

The viscosity, volume, and pH of media are other important factors. Low viscosity and acidic medium contained lower bacterial count led to improved microbial reduction rate during US processing. Because higher viscosity leads to a higher cavitation threshold and, consequently, a more energetic bubble collapse [29]. As the target level of US pasteurization is to decrease 5 Log of pathogenic microorganisms, the initial microbial content of food products is critical [43]. The characteristics of microorganisms, including their cell wall, size, shape, spores, and growth phases, are also critical [16].

Studies have also shown that the food matrix plays an influential role in inactivating microorganisms by US. Lactose has been suggested to increase the resistance of microorganisms to the US by stabilizing bacterial membranes and accumulating compatible solutes [37]. Fats also play a protective role for fungus because they can increase the resistance of fungus to inactivation treatments either by decreasing the water activity (aw) of the system or by dehydrating cells immersed in the lipid phase of the system [2].

## 5 Effects of US on food structure and properties

Although the ultrasound technique has numerous advantages over conventional methods and has been widely used in the food industry, this technique can have adverse effects on food quality, micronutrients, and macronutrients. Due to the high temperature and pressure conditions caused by acoustic cavitation, it can cause the accumulation of radicals, which can interact with food compounds and cause the degradation of these compounds. The effects of US on the texture, sensory, and nutritional properties of food depend on the US conditions (power, intensity, energy density, duration) and food properties (solid, liquid, semi-solid, and viscosity) [6]. The effects of US on the structure and nutritional properties of food are mostly related to the phenomenon of cavitation. The implosion of cavitation bubbles causes energy to accumulate in hot spots, creating extreme temperatures of up to 5000 K and pressures of up to 5000 atm, which cause high-shear-energy waves and high turbulence in the cavitation region [34].

Many studies have been conducted in this field In the US processing of green asparagus during cold storage (40 kHz frequency and 360 W power for 10 min), critical parameters related to product quality, including total soluble loss, ascorbic acid, chlorophyll levels, and total phenolic components, are evaluated. The loss of total solids, which reflects plant tissue respiration, is higher in treated samples due to the inactivation of metabolic enzymes. However, treated samples show lower levels of ascorbic acid during storage time because of oxygen elimination, resulting in lower ascorbic acid degradation and destruction of some enzymes involved in this reaction. A similar trend was also observed in the chlorophyll content of products, wherein cavitation reduced enzyme activity [48]. However, other studies showed different results and reported that ultrasound does not have a negative effect on the quality and nutritional properties of the products. For example, tomato juice's pH, lycopene content, and vitamin C content did not show significant differences between control and sonicated juices until 10 days in cold storage conditions. The amount of lycopene was significantly higher in samples treated with ultrasonic probe (set at 750 W, 40 W/cm^2^ intensity, 20 kHz, 19-mm diameter probe), for 2 min and lower in samples treated with 10 min of US. In addition, the vitamin C content in samples treated with US for 10 min was considerably reduced. The color changes were dependent on the wave intensity. Furthermore, some microstructural differences were observed among control and treated samples [45]. With reduced exposure time, a cell cluster was observed compared with control samples; however, with prolonged exposure time (10 min), the cell structure of juice remained unchanged [45].

In another study performed by Alenyoregea et al. regarding the efficacy of sweep frequency US on Chinese cabbage, quality parameters such as chlorophyll content, texture, color, and enzyme activity were measured. The color of samples treated with 40 kHz sweep frequency US showed higher variation, probably due to cavitation mechanisms. The best color without significant statistical differences during the storage period (3 days) was reported. The firmness and stability of sonicated cabbage, which are indicators of texture quality in fruits and vegetables, were also improved due to the inactivation of associated enzymes, including pectin methylesterase and polygalacturonase, which are responsible for the depolymerization of molecular cell walls. The chlorophyll content of Chinese cabbage decreased marginally during the whole preservation time without significant alteration between processed and unprocessed samples [3].

In another study, Fan et al. [14] reported that US (power of 400 W, intensity of 226 W/cm^2^, and 15 mm diameter probe) reduced the weight loss, total soluble solids, firmness, color variation, ascorbic acid, and flavor degradation of modified atmospheric packaged fresh-cut cucumbers (20 kHz, 10 min). In addition, the water mobility decreased, and the integrity of cell walls during storage was better preserved than control samples [14]. The properties of food products treated with US vary according to the processing conditions applied. However, various studies have observed better quality of food components treated with US than traditional decontamination methods [43].

The conformation and structure of proteins may be affected by the US process. These changes depend on the USlower, duration, and temperature. The protein chain is opened by applying US, and hydrophobic and hydrophilic groups can be exposed, related to increased protein solubility and foaming properties [19]. Sheng et al. [41] reported that after applying US at 360 W with a 10 mm diameter titanium probe, the foaming properties of egg whites increased by about 4.9 times compared with the control group [41]. In addition, US treatment can cause the formation of dimers or polymers in unacceptable conditions. Therefore, US treatment's duration, power, and temperature should be optimized to preserve proteins' biological and technological properties and inactivate microorganisms [19].

## 6 Recent research regarding the application of US for fungal decontamination

The investigation regarding the industrial use of US treatment on different categories of food, including dairy, cereal products, beverage, fruit, and vegetable, indicated various results on the food properties at various conditions. Thus, finding the optimal parameters for each product is important. The findings of recent investigations regarding the application of US for fungal decontamination in food products are presented in Table 1. Grains are a critical source of food products in the cereal industry that fungi and pests can easily infect, and the detection of mycotoxins is vital to promote health. Rudik et al. investigated the effects of US with intensity from 0.3 W/cm^2^ to 1.5 W/cm^2^ and frequency range of 22–35 kHz on wheat disposal. The results of mycotoxin evaluation indicated that fungal growth after US treatment with 24–26 kHz at 1 W/cm^2^ was significantly reduced, and up to 98% of microorganisms were inactivated [39].

**Table 1:** Recent studies (last four years) on the Ultrasound processing of food products for reduction of microorganisms’ contamination.

Type of food	Type of Processing	Experimental conditions	Type of Microorganism	Main outcomes	Reference
Strawberry Fruit	Bath Ultrasound + Aqueous Ozone	Frequency: 40 kHz Treatment time: 3, 6 and 9 min Ozone flow rate ≥3.3 mg/min for 125 g strawberry/L water	Mesophilic aerobic bacteria and fungi	~2 log CFU/g reduction in mesophilic aerobic bacterial count by US and ozone / combination of US and ozone extended storage time before fungal decay	[28]
Tomato juice	Ultrasound Probe	Intensity: 28 and 40 W/cm^−2^ Treatment time: 2, 5 and 10 min Sonication temperature: 37–52 °C product storage time: 1, 4, 7 and 10 days	Mesophilic aerobic bacteria, Lactic acid bacteria. Coliforms. Yeast	2 min treatment was insufficient for microbial decontamination. 5 min of sonication with 40 W/cm^2^ intensity: (Mesophilic aerobic bacteria: 4.3 log10 CFU/g reduction) (Lactic acid bacteria: <1 log10 CFU/g reduction) (Coliform: was detected only on fourth storage day (1.7 log 10 CFU/g) and seventh day (1.8 log 10 CFU/g)) Yeast: <10 CFU/g 10 min of sonication with 40 W/cm^2^ intensity: Mesophilic aerobic bacteria: reduced by 2.8–5.4 CFU/g Other microorganisms were reduced to below the limit of quantification	[45]
Fresh Tumeric	Electrolyzed water & ultrasound	Electrolyzed water from 5% NaCl US frequency: 43 kHz treatment time: 10 min	*E.coli*, Mesophilic aerobic bacteria, yeast and molds	EO 200 mg/L with US at 43 kHz had high value of log reduction on all microorganisms	[52]
Wheat grain	Ultrasound– anolyte medium	US frequency: 22–35 kHz US intensity: 0.3 W/cm^2^ to 1.5 W/cm^2^	Mesophilic aerobic bacteria and fungi	Mesophilic aerobic bacteria and Fungi numbers decreased significantly, the use of US with anolyte has shown a synergistic effect.	[39]
Green asparagus	Bath Reactor Ultrasound	Frequency: 40 kHz Power: 360 W Treatment time: 10 min stored for 20 days at 4 ˚C	Mesophilic aerobic bacteria/ mold and yeast	Mesophilic aerobic bacteria, mold and yeast in the 12th and 16th day of storage decreased significantly	[48]
Chinese cabbage	Sweep ultrasound	Frequency: 28, 33, 40, and 68 kHz Treatment time: 10 min	Mesophilic aerobic bacteria, yeast and molds	The significant reduction for mesophilic bacteria was 0.24–1.83 log CFU/g And for yeast and mold was 0.15–2.26 log CFU/g	[3]
Cucumber	Ultrasound Probe	Frequency: 20 kHz Treatment time: 5, 10, and 15 min	Mesophilic aerobic bacteria and yeast and molds	Most significant reduction after 10 min processing	[14]
Orange Juice	Thermo-ultrasound processor with S3 probe	Treatment temperature: 50 ˚C Frequency: 24 kHz	*S. cerevisiae*	About 1.5 log reduction in *S. cerevisiae*	[40]
Lettuce	Electric current & Ultrasonic bath	Low intensity electrical current: 0.2, 0.8, and 1.4 A Frequency: 24 and 40 kHz Treatment time: 2, 4, 6, 8, and 10 min	Coliform and yeast and molds	Coliforms: the individual use of the ultrasonic bath at 40 kHz frequency was more effective than electro-sonication treatments. Yeast and mold counts: individual electrical treatment was more effective	[21]
Alfalfa and mung bean sprouts	Ultrasound probe form	Frequency: 26 kHz Amplitude: 90 µm Power: 200 W	*Salmonella, E. coli*	Alfalfa: 1.40, 1.06 log CFU/g reduction on *Salmonella* and *E. coli* respectively/ mung bean sprouts: 1.89 and 1.23 log CFU/g reduction on *Salmonella* and *E. coli* respectively	[53]

Studies have shown that using a liquid medium for US treatment increased the efficiency of the process. In other words, products that contain more water, such as fruits and vegetables, are more affected by the US [27]. Because US treatment of water makes hydroxyl, hydrogen, and hydrogen peroxide radicals act as initiators to break covalent bonds in mycotoxin molecules. In addition, using anolyte solution combined with US can synergistically decrease the number of microorganisms [30]. Also, the high US propagation in solid foods causes more energy and acoustic power to be transferred to food and increases the fungicides power [46].

The initial concentration of mycotoxins plays a vital role in the effectiveness of US in mycotoxin decontamination. Liu et al. [26] reported that the rate of DON degradation decreased from 50% to 37% with increasing DON concentration from 2000 µg/L to 4000 µg/L by ultrasound (A 550 W US processor with a 13 mm probe at a constant frequency of 20 kHz for 40 min) [26]. With increasing concentrations of mycotoxins, there will not be enough free radicals to degrade all DON. Therefore, the power of US and treatment time should be increased with increasing concentrations of mycotoxins.

## 7 Combined approaches for fungal inactivation and US in food and beverage

US is a promising technology for bacterial and fungal inactivation of a wide range of food products. Combining US with other technologies can save costs and increase its effectiveness against resistant microflora. The effect of US on fungi inactivation depends on various factors such as US power, operation time, types of fungi, the volume of food being processed, and food composition [13]. Hence, specific US frequencies should be applied against different yeasts and molds. Therefore, the US is usually used with other treatments such as heat treatment, UV, pulsed electric field, and ozone [5].

In the study of Maryam et al., strawberry samples were treated with ozone (flow rate ≥3.3 mg/min for 125 g strawberry/L water) and US (40 kHz at 100 W) for 1, 2, or 3 min and evaluated under storage at 2 ± 0.5 °C for 12 days (relative humidity 95%). In addition, fungal decay was postponed by 4 days, and the shelf life of strawberries under cold preservation was increased by 6 days [28]. In another study, *Saccharomyces cerevisiae* in natural orange juice was inactivated using thermo-US (24 kHz; 105 µm; 33.31 W/mL at 50 °C) and cinnamon leaf essential oil (650 ppm). *Cinnamomum spp*. is widely used as a flavoring agent due to its inhibitory effects against pathogenic bacteria, fungi, viruses, and spoilage organisms. The results showed that the combination of essential oil and US at 50 °C (2.52 log reduction) was effective for orange juice decontamination compared with untreated samples as the control group (1.32 log reduction) [40]. In another study, US with the power of 400 W were used for the process of loquat fruit while immersed in per acetic acid (0.4% w/w) at 20 °C for 6 min. Evaluation of loquat fruit stored for 12 days under 95 % relative humidity showed that this method inactivated enzymes and decreased products' decay and browning index [24]. Kilicli et al. [21] applied three electric currents (0.2, 0.8, and 1.4 mA) and two ultrasonic processes, including ultrasonic bath (40 kHz) and ultrasonic processor (24 kHz) for 2, 4, 6, 8, and 10 min to pathogen decontamination of lettuce. The results represented that the use of electric current and ultrasound individually inactivated the microorganisms. However, the destructive effect of ultrasound was limited. Also, combining these two methods did not indicate significant differences and sometimes resulted in antagonistic impacts.

In general, US treatment can increase decontamination of microorganisms when combined with other technologies, including chemical and heat treatment. Also, if the inactivation of all pathogen microorganisms by using ultrasound is targeted, further negative effects on food quality (structure, flavor, micro, and macro-nutrients) are possible. Therefore, the combined use of ultrasound with other methods reduces the damage caused by ultrasound.

## 8 Perspectives and conclusion

The most effective methods for inactivating mycotoxins in food should not produce any toxic compounds in food and should not have a negative effect on the sensory and nutritional properties of food. Ultrasounds are considered a non-thermal decontamination method in the food industry. It can inactivate some microorganisms and enzymes, and if the time, frequency, and intensity of ultrasound are optimized for each product, this method does not significantly affect the quality and sensory properties of the food products. This phenomenon is due to the cavitation mechanism created by wave power. The treatment conditions applied (amplitude, intensity, pressure, and duration of sonication), types of food components, and properties of microorganisms (growth phase, size and shape, wall composition, and thickness) are critical parameters influencing the effectiveness of US. Therefore, US can be considered an effective method in decontaminating mycotoxins due to low processing temperature, less energy consumption, and less nutrient and flavor loss. However, the simultaneous use of US with thermal or non-thermal methods has shown some synergistic and antagonistic effects on fungal inactivation and mycotoxin decontamination. Therefore, further studies are needed to determine which method is most compatible with the US.

## CRediT authorship contribution statement

**Motahareh Hashemi Moosavi:** Visualization, Methodology, Writing - original draft, Investigation, Writing - review & editing. **Amin Mousavi Khaneghah:** Conceptualization, Writing - review & editing, Supervision. **Fardin Javanmardi:** Visualization, Methodology, Writing - original draft, Investigation, Writing - review & editing. **Milad Hadidi:** Writing - review & editing. **Zahra Hadian:** Writing - review & editing. **Shima Jafarzadeh:** Writing - review & editing. **Elcin Huseyn:** Writing - review & editing. **Anderson S. Sant'Ana:** Conceptualization, Writing - review & editing, Supervision, Project administration, Funding acquisition.

## Declaration of Competing Interest

The authors declare that they have no known competing financial interests or personal relationships that could have appeared to influence the work reported in this paper.
